# Improving Infant Mental Health: A Pilot Study on the Effectiveness, Acceptability and Feasibility of Eye Movement Desensitization and Reprocessing (EMDR) Storytelling in Infants With Post-traumatic Distress After Medical Procedures

**DOI:** 10.1177/01632787241268176

**Published:** 2024-10-23

**Authors:** Eva S. Potharst, Petra Holtkamp, Lily Walliser, Agnes H. Dommerholt, Maartje E. N. van den Heuvel, Indra Spierts, Marija Maric

**Affiliations:** 1 1234 Academic Outpatient (Child and Adolescent) Treatment Center of the University of Amsterdam, The Netherlands; 2 1234 University of Amsterdam, The Netherlands; 3 Infant Mental Health Center OuderKindLijn, The Netherlands; 4 Medical Pedagogical Center ‘t Kabouterhuis, The Netherlands; 5 10215 Hospital OLVG, The Netherlands; 6 EMDR Zuid, The Netherlands; 7 Psychological Practice Oog, The Netherlands

**Keywords:** infants, post-traumatic stress disorder (PTSD), post-traumatic distress, eye movement desensitization and reprocessing (EMDR), EMDR storytelling, intervention, single case experimental design

## Abstract

Although the prevalence of symptoms of post-traumatic stress disorder (PTSD) in infants and young children is similar as in older age groups, and PTSD intervention is as important in this age group, research on PTSD-treatment in infants is very scarce. Eye Movement Desensitization and Reprocessing (EMDR) Storytelling is a trauma-focused treatment that is being used by clinicians for infants with PTSD-symptoms. The aim was to assess the feasibility, acceptability and initial indications of effectiveness of EMDR Storytelling for infants aged 3–24 months with PTSD-symptoms after medical procedures. We included 6 infants and administered personalized items to assess PTSD-symptoms during the baseline, intervention and follow-up phase on a day-to-day basis. Furthermore, we measured PTSD-classification and symptoms at three and four measurement points, respectively. The data was analysed visually and quantitatively. EMDR Storytelling was shown to be feasible and acceptable for all participating families. Parent- and therapist-report showed that four out of the six infants included in the current study showed a clear reduction over time in PTSD-classification, -symptoms, and daily measured PTSD-symptoms. The results concerning the other two infants were mixed. Attention should be paid to cognitive (language) as well as interactional (infant-parent) mechanisms potentially underlying the benefits of EMDR Storytelling.

## Introduction

Early life stress is a risk factor for poorer long-term emotional, behavioral and cognitive development (Smith & Pollak, 2020; VanTieghem & Tottenham, 2017). Especially family-related factors such as domestic violence have been studies extensively as early life stress factors (Vu et al., 2016). Another circumstance that may evoke high levels of stress in young children is hospitalization (D’Agata et al., 2017). An international systematic review study showed that 4.6%–9.0% of all infants need to be hospitalized after birth (Lebreton et al., 2020). These infants regularly undergo painful and invasive medical procedures, such as (repeated) blood tests, endotracheal intubation, and insertion of peripheral lines. There is growing awareness in the field of Neonatology that medical procedures and repetitive pain can have negative consequences for infant neurocognitive development (Grunau et al., 2006). Much less attention goes to the possibility that as a consequence of such procedures and experienced pain, as well as the often accompanying separation from the parent, an infant can develop post-traumatic stress symptoms (Lakatos et al., 2019; Perry & Pollard, 1998).

Van der Kolk (1997) defines trauma as “the result of exposure to an inescapably stressful event that overwhelms people’s coping mechanism” (p. 243). In 1995, Scheeringa et al. published a study on the diagnosis of PTSD in infants and young children, and concluded that the fourth edition of the Diagnostic and Statistical Manual (DSM-IV) criteria were not reliable and valid enough for this age group. On the basis of 20 case reports of severely traumatized infants, they made a checklist of manifestations of symptoms that were more developmentally sensitive, that improved the correct diagnosis of PTSD in infants and young children. Examples of the new diagnostic criteria were: episodes with objective features of flashback or dissociation, exaggerated startle response, night terrors, social withdrawal, and restricted range of affect. These criteria were the basis of the criteria for Traumatic Stress Disorder, that was included in the first edition of a broad and developmentally based diagnostic manual for infants and toddlers, the Diagnostic Classification of Mental Health and Developmental Disorders of Infancy and Early Childhood (DC:03, Zero-to-three, 1994). The availability of the DC:03 improved the recognition of mental health problems in infants by mental health care workers, and made it possible to study these in a more standardized way.

However, about 15 years after this development, De Young, Kenardy, and Cobham (2011) concluded that although clinical and academic attention for several mental health problems in infants had increased, trauma in infants and young children was still a largely neglected problem. In a review on this topic, they described what had become clear in the previous years, namely that due to their limited coping skills and the phase of development they are in, young children seem to be particularly vulnerable to experiencing post-traumatic symptoms after adverse events. Furthermore, they concluded that young children have the developmental capacity to be able to experience psychopathology following trauma, and that untreated symptoms of trauma can develop into risk of chronic and debilitating symptom trajectories and can negatively influence development. Since then, a large step forward has been taken by the inclusion of developmentally appropriate PTSD criteria in the DSM-5. Research using these criteria, showed that the prevalence of PTSD in infants and young children is similar as in older age groups (De Young & Landolt, 2018).

Most studies on PTSD in young children focus on children older than 12–24 months (e.g. Graf et al., 2011; Meiser-Stedman et al., 2008; Scheeringa & Zeanah, 2008), while only a few included infants younger than 12 months (e.g. DeVoe et al., 2006; Graf et al., 2013). Medical trauma in infants in their first year of life has been described in the context of childhood cancer and prematurity. Graf et al. (2013) assessed PTSD in 48 children between the age of 8 months and 48 months with childhood cancer. They found that about one fifth of the children met full criteria of PTSD, and about two fifth of the children met the criteria of partial PTSD. A case study by Roy and Russell (2000) described post-traumatic symptoms in a boy of 5 months old, who was diagnosed with cancer at the age of two weeks, and was hospitalized several times, during which he underwent invasive medical procedures. Parents observed changes in their child’s behavior that may have been related to medical trauma, such as spontaneous awakenings during the night, after which he would cry uncontrollably for an hour or more, difficulty to be soothed after noninvasive procedures, social withdrawal, and an exaggerated startle response.

Maronay (2003) posed the hypothesis that the psychological, emotional, and behavioral problems that are associated with preterm birth may partly be caused by chronic stress and trauma during hospitalization at the Neonatal Intensive Care Unit (NICU). In a review, Maronay (2003) described that chronic stress and trauma may have larger negative consequences in infancy than in later periods of life, as such an important development in the brain takes place in this period. Neurological structures and pathways may develop differently to deal with the chronic stress and trauma and may make the brain more sensitive to subsequent stress not only during the hospitalization period, but also later in life (Perry et al., 1995). Maronay explains that “theoretically, as a premature infant grows, he/she may not be able to distinguish, on a subconscious level, between the here and now of a stressful event and the past events of the NICU due to the atypical development of his/her brain.” Symptoms of trauma in children, such as impulsivity, attention problems, dysphoria, emotional numbing, avoidance behaviors, fears, sleep problems, aggression, and withdrawal are similar to the problems that many preterm born children face when they grow up (Maronay, 2003). D’Agata et al. (2017) also suggest that not only the early developmental stage at birth, but also traumatic NICU experiences may serve as an extra risk factor for developmental problems in preterm born children. D’Agata et al. (2017) proposed a new concept to describe the intertwined and cumulative early life experiences of stress, parental separation, and pain, which may lead to alterations in short- and long-term neurodevelopmental and physiological responses: Infant Medical Trauma in the Neonatal Intensive Care Unit (IMTN).

Not only experiences in the NICU (summarized as stress, parental separation, and pain by D’Agata et al., 2017) are risk factors for later symptomatology. Also parental factors play a role in the development of PTSD symptoms in infants. In infants and young children with cancer, more severe maternal PTSD increased the risk for full or partial PTSD in the children. In infants and young children with burns, the number of PTSD symptoms in the children were also positively associated with maternal PTSD, and negatively associated with a higher quality of family relations (Graf et al., 2011). The same association between child and maternal PTSD symptoms was shown in a study on young children who witnessed intimate partner violence (Bogat et al., 2006). Scheeringa and Zeanah (2001) described this phenomenon as relational PTSD: “the co-occurrence of posttraumatic symptomatology in an adult caregiver and a young child when the symptomatology of one partner, usually the adult, exacerbates the symptomatology of the other.”

Winnicot (1964) wrote “there is no such thing as a baby.” He meant that a baby cannot be alone; someone is needed to take care of the baby, both physically and emotionally. A baby is dependent on caregivers not only for physical care, but also for support in maintaining and regaining emotional stability after stress. The latter happens through co-regulation, which is the bidirectional linkage of oscillating emotional channels between a mother and child that contributes to emotional and physiological stability for both (Butler & Randall, 2013). In a co-regulation relationship between a baby and a parent, regulation abilities of both the baby and the parent are important factors. A baby with post-traumatic stress, who, as a consequence, suffers from regulation problems, will make a greater appeal on his parents for co-regulation. Also, a post-traumatic stress reaction in the baby will increase a parent’s tendency to perceive the child as vulnerable after serious illness or preterm birth (Allen et al., 2004), and to worry about their child. On the other hand, when the parent has problems in self-regulation, he will be less emotionally available to support the baby with his regulation. Therefore, parental psychopathology, such as PTSD, is a risk factor for co-regulation problems between parents and a baby. Infant illness, hospitalization and medical procedures, however, are not only a risk factor for post-traumatic stress in children, but also in parents (Balluffi et al., 2004; Schecter et al., 2020). This may mean that children with medical trauma on the one hand have a relatively high need for support in regulation, while the parents’ emotional availability on the other hand may be negatively affected because of post-traumatic stress of the parent. This may lead to a vicious cycle, and a continuation of co-regulatory difficulties and PTSD symptomatology.

Because of the dependency of the baby on his parents, and the intertwined nature of post-traumatic symptoms of parents and child, and co-regulatory problems between them, the involvement of parents in treatment of the infant is essential. D’Agata et al. (2017) describe the importance of supporting parents in the development of a supportive relationship with their children, and also the importance of creating language for parents to describe the experiences their infant had during the hospitalization. The infant behavioral assessment and intervention program, implemented in the Netherlands to teach parents of very preterm born children to be responsive to their infant and support their regulation, showed positive developmental outcomes for the children (Verkerk et al., 2012). Other studies focused on preventing or reducing PTSD symptoms in parents whose infants had been hospitalized in a NICU by teaching parents coping and self-regulation skills (Shaw et al., 2013; Horwitz et al., 2015; Kraljevic & Warnock, 2013). Although there is evidence on the effectiveness of parenting programs focused on improving the co-regulatory parent-child relationship, and prevention or treatment of parental trauma on the other hand, in some cases it may be needed that a more direct treatment of the infants’ trauma is offered. Psychologists (such as IS and PH) in the Netherlands observed that some infants kept showing severe symptoms of trauma, also after parental support in improving the co-regulation relationship with their child, and trauma-focused treatment for the parents.

Literature on infant trauma treatment is scarce. Child-Parent Psychotherapy, an Infant Mental Health informed psychodynamic, dyadic psychotherapy, is described as a potential treatment for parents and infants during their stay at the NICU (Lakatos et al., 2019). This form of dyadic psychotherapy is not only focused on improving the parent-child relationship, but also improve reflective functioning of the parent, which supports the development of a narrative on the traumatic events, that can be shared between parents and child. Weatherston and Browne (2016) describe a similar Infant Mental Health informed treatment for parents and high-risk infants in an outpatient setting, which can be offered in the period after hospitalization.

Interventions for traumatized children at toddler age are mostly based on cognitive-behavioral therapy (CBT), but are also preferably dyadic, and take into account the relational context of posttraumatic symptomatology (Rachamim et al., 2017). Four toddlers with medical trauma and their parents were offered prolonged exposure therapy, which included psychoeducation, recounting of the traumatic events by both the child, the parents and the therapist, and in-vivo exposures (Rachamim et al., 2015). Toys and play were used in the different phases. Post-traumatic stress symptoms in both the toddlers and parents decreased. In a pilot study on treatment of PTSD in 12 preschoolers, effectiveness of dyadic exposure therapy was compared with dyadic client-centered therapy (Rachamim et al., 2021). Only dyadic exposure therapy was shown to be effective in reducing child and maternal symptoms of PTSD. In a randomized controlled trial among 64 toddlers and preschoolers with PTSD, trauma-focused CBT was shown to be more effective than being on a waitlist (Scheeringa et al., 2011). Rachamim et al. (2017) underscored the importance of making use of a case conceptualization prior to a trauma-focused CBT for toddlers. They suggested to use a case conceptualization model that is developmentally appropriate, which means that it should include aspects of the parent-child relationship, and the way that the child’s and parent’s cognitions, emotions and behaviors mutually influence each other. In case of medical trauma, they advised to always attend to PTSD (symptoms) in both the children and the parents, parental coping and parental functioning, possible parental overprotection, and possible consequences that the trauma may have had for the quality of child-parent attachment (Rachamim et al., 2017). Making use of a similar case conceptualization could also be a prerequisite for a trauma-focused therapy for infants and their parents. Thus, there is promising first evidence that post-traumatic stress symptoms in toddlers are treatable. However, from the research, we do not know yet whether such effects can also be obtained below 24 months, whether this can be attained with a brief manualized intervention, and how such an intervention could be offered, given the developmental stage.

Surprisingly, none of the above studies have included EMDR with toddlers, even though EMDR has been found to be effective at reducing symptoms of PTSD in older children, adolescents and adults (Hoogsteder et al., 2022; Lewey et al., 2018; Wright et al., 2024). For the treatment of trauma in older children, Eye Movement Desensitization and Reprocessing (EMDR) has been shown to be efficacious, also in comparison with CBT (Rodenburg et al., 2009; Moreno-Alcazar et al., 2017; Lewey, 2018). To make EMDR treatment acceptable for the treatment of traumatic distress in very young children and infants, Lovett (1999) adjusted the standard EMDR protocol, resulting in EMDR Storytelling. EMDR Storytelling consists of two phases: (1) a preparation phase in which parents and a therapist write a story about the traumatic event(s) from the perspective of the child, and (2) an actual treatment phase, in which the story is read to the child who is being held by one of the parents, while the infant’s working memory is being taxed by EMDR techniques. Since Lovett (1999) made these adjustments to the treatment protocol for infants, the treatment protocol has been used by clinicians. The EMDR Storytelling was translated into Dutch by De Roos and Beer (2017). Three members of our project group (PH, AD and IS) use EMDR Storytelling for over 10 years with young children and infants in line with Lovett’s protocol, and its Dutch translation. They have observed that the trauma-related regulation problems as reported by their parents diminish or disappear after one or two sessions. These results are in line with other anecdotal evidence showing positive effects of EMDR Storytelling in previously hospitalized infants (Lovett, 1999; Went, 2014). As to date, no systematic investigations of the effectiveness of EMDR Storytelling exist.

What is also missing in the scientific literature, is a clear range of the age of the children that EMDR Storytelling is suitable for. Especially the lower age limit is of interest, as EMDR Storytelling partly relies on language comprehension of the child. Infants start to understand words at the age of 6–9 months, and this ability rapidly increases in the second year of life (Bergelson & Swingley, 2015), but language development starts well before this age (Saletta & Windsor, 2018). In our project group, clinical experience of successful treatment with EMDR Storytelling exists with infants as young as a few months, who may have not yet acquired the ability to comprehend words. In infants of this age group, and also in older infants, it is hypothesized that traumatic memories are not triggered by words and sentences (only), but also by other elements, that are used during the treatment. Trauma related stimuli to activate the trauma memory are used in the EMDR Storytelling therapy. For example, medical objects are shown during the reading of the story, audio-files with sounds of the intensive care are played, and body-parts of the baby that were involved in the traumatic events are touched. Furthermore, it is assumed that while the story is being read to the infant by one of the parents, the other parent, who is holding the infant, is emotionally and physiologically affected by the story, which subsequently affects the infant. Research has shown that infants’ stress level can be regulated by being held by a parent (Kommers et al., 2018). Microanalysis of mother-infant communication has shown that subtle parent behavior such as facial expression or vocalisations (Beebe & Steele, 2016) and maternal prosody (Spinelli et al., 2017) influence infant (dys)regulation. During storytelling, it may be that when the parent experiences emotions, tension in the infant’s body also builds up, which is then diminished by the taxation of the working memory of the infant, which is the other element of an EMDR Storytelling session. Therefore, EMDR Storytelling may be suitable for infants who do not yet, or minimally understand spoken language.

Regarding the taxation of working memory: in a recent state of the science article on EMDR, this has been identified as the hypothesized mechanism of action of EMDR for which most evidence is available (de Jongh et al., 2024). The idea is that EMDR involves a dual focus of attention or ‘dual’ taxation (Wadji et al., 2022), namely the combination of the retrieval of the traumatic memory and another ‘task’ that is demanding capacity of the working memory. It is hypothesized that because the ‘extra’ task is limiting the capacity of the working memory, the working memory is not capable to retain all elements of the memory, and the intensity of recall of the traumatic memory is decreased (de Jongh et al., 2024). Because infants are not able yet to follow instructions for a working memory taxing task, the therapist makes sure that she is offering activities to the baby that are demanding for the working memory. It has been shown that matching the taxation tasks to the recalled memory (e.g. a visuospatial task when treating a visual memory) has the largest effect, but that nonmatched tasks (such as a verbal task when treating a visual memory) also appear to be effective (Matthijssen et al., 2019). Because the traumatic memories of infants with a history of medical procedures are hypothesized to involve bodily sensations, the task or activity that the baby is being offered should preferably be a task that also involves physical sensations. Therefore, gently tapping body parts of the infant (for instance the knees of the baby) has been chosen as the ‘task’ that taxes the working memory.

To the best of our knowledge, no other studies exist on the effectiveness of EMDR Storytelling in infants with medical trauma. As indicated before, psychological and psychosocial care for parents is already an integral part of the follow-up program in the hospitals. As our end users observe in their daily practice, suitable, evidence-based interventions directed directly at post-traumatic distress of the infant are non-existing, but at the same time, highly needed. In the infant mental health clinical practice, EMDR Storytelling is already used with families with an infant with PTSD symptoms.

Therefore, the aim of this pilot study was to assess the feasibility, acceptability, and initial indications of effectiveness of a 2-sessions EMDR Storytelling intervention for infants aged between 3 and 24 months. Given the relative innovative character of this study and intervention, and the potential heterogeneity in treatment effects between participants (Maric & Kok, 2023), single-case methodology was implemented to investigate our research questions. Other reasons for choosing a single case experimental design (SCED) were the limited number of infants that we expected to include, which would limit power in a more traditional design such as a randomized control, and ethical consequences of a randomized design, in which some infants would have been randomized to a condition in which they would not have been offered trauma treatment. Infant trauma symptoms were measured daily using personalized items during a baseline, intervention and follow-up phase, and additionally by a parent-report questionnaire and a therapist-report classification at three and four measurement points, respectively. We expected to find a stronger decrease in the daily measurements of trauma symptoms in the intervention phase and follow-up phase, as compared to the baseline phase. We also expected improvement in parent-reported infant PTSD-symptoms and therapist-reported infant PTSD-classification at posttest and three-week follow-up.

## Methods

### Design

The SCED method was used in this pilot study. SCED is widely recognized for its potential to support investigation of novel interventions prior to investigation in large-group studies (Maric & van der Werff, 2020). We implemented an ABC SCED with baseline phase A, followed by an intervention phase B, and follow-up phase C. A within-subject comparator (intervention phase B vs. baseline phase A in each participant) was included. Personalized items were constructed on the basis of the child’s symptoms assessed prior to baseline phase A, and administered daily during phase A, B, and C. Before and at the end of each phase (at T0, T1, T2, T3) questionnaires were completed by the parents. Furthermore, a classification according to the DC: 05 of the child’s trauma symptoms was made at T0, T2 and T3.

The baseline phase, intervention phase and follow-up phase were designed to be of similar length (three weeks of daily measurements), so that (i) the phases could be compared easily; (ii) this number of observations seemed sufficient for the planned analyses, and (iii) this design was considered feasible for all involved parties. The baseline phase is meant to offer comparison, as here there is a logging of the infant’s behavior that is potentially PTSD-related, as represented by the daily items, while there is no explicit therapy or intervention directed at this behavior. A ‘care-as-usual' was not standardized here; it consists of the individual handling of the baby by his family. The baseline period of three weeks was based on the time that was needed for the preparations for the intervention (writing the story to be read for the infant), and to make sure that even with missing data points, we would be able to gather a representative, stable sampling of the infant’s behavior (Smith, 2020). For this, a minimum of 5 data points were necessary, but baseline periods with more data points may be more representative and therefore increase validity (Smith, 2020). In practice, the period in which the daily items were administered was sometimes shorter, because in some cases the creation of the daily items took more time than expected. However, the minimum of 5 data points was reached for all participants. The baseline period of infant 1 was a lot longer than planned (46 measurements). The reason for this was that despite the negative screening of PTSD in the mother, during the writing of the story, it appeared that her PTSD symptoms were more severe than expected, and that she needed PTSD treatment herself before it was possible to proceed with the intervention phase in which she needed to read the story to her infant. For the other infants, the number of daily measurements in the baseline period ranged from 7 to 16.

The intervention period was also designed to last three weeks. This period consisted of and EMDR sessions on day 1 and ideally on day 8, and a period of two weeks afterwards which was needed for integration and consolidation of the treatment effect. This period of two weeks was based on clinical experience of the therapists in the research team. Due to illness, quarantine rules related to COVID-19, family vacation, the second session was postponed for infant 4, which resulted in a prolonged intervention period of 45 days. For the other infants, the number of daily measurements in the intervention period ranged from 14 to 19. The number of measurements in the follow-up period ranged from 13 to 23.

### Participants and Procedure

Infants aged 3–24 months with a history of hospitalization at the OLVG hospital in Amsterdam, and potentially traumatic experiences related to painful medical procedures [for example (repeated) blood tests, endotracheal intubation, insertion of peripheral lines and surgery] were eligible for this study. Six infant within the age range 4–23 months, and at least one of their parents/caregivers were included in this study. Inclusion criteria were: (i) according to the DC: 0-5 diagnostic interview (2016): A positive score on Posttraumatic Stress Disorder on Axis I (Clinical disorders), a negative score on level 3 and 4 of Axis II Relational context (disrupted relationships between parent and infant should be absent); (ii) the infant experiences regulation problems such as crying, sleeping and eating difficulties, clinging to the parents, as indicated by at least one item of the ‘Infant Trauma-related Stress Symptoms Questionnaire’. The DC:05 and Infant Trauma-related Stress Symptoms Questionnaire are described in more detail under the heading ‘Outcome measures’. Exclusion criteria were: (i) Parents having PTSD themselves, and (ii) parents who cannot commit to the research.

The enrollment process for the study was as follows. At the OLVG hospital in Amsterdam, families who were admitted to follow-up care were screened, and referred to Infant Mental Health center OuderKindLijn (OKL) for EMDR Storytelling, in the case that the infant showed trauma symptoms. If at least one of the parents had PTSD themselves, the parent was referred for treatment first. At OKL, families were invited for an intake, in which a case conceptualization was constructed, a classification of symptoms was done according to the DC: 05, and study inclusion and exclusion criteria were checked. If families met these criteria, they were asked to consent for participation in the study. In case EMDR Storytelling was not indicated, families were referred to another appropriate intervention at OKL.

After the parents gave signed informed consent, parents completed the first set of questionnaires (T0). The personalized daily items were created by the parents, a psychology student doing a research internship (LW in some of the cases), and one of the researchers on the project (EP), in some cases with input from the therapist (PH in some cases). The items were based on the high scoring items of the questionnaire that was used to measure trauma symptoms, on observation of the behavior of the infant by the parents, and hypotheses about which behaviors may be related to the traumatic events. Subsequently, the baseline daily measurements started (phase A). During the baseline phase, parents wrote the story of the traumatic events from the perspective of the infant, with the help of the therapist. In a session with the therapist, parents received instructions for writing the story, and then wrote the story at home. Mostly, another sessions was used for correction of the story, and for preparing the actual EMDR with the infant. A few days before the first EMDR session with the infant, parents completed T1. Then, two EMDR Storytelling sessions took place, ideally with one week in between sessions. At the day of the first EMDR session, phase B started. This phase lasted until two weeks after the second session. After parents completed T2 and a new classification of the infant’s trauma symptoms was made according to the DC: 05, the follow-up phase started (phase C). This phase lasted for three weeks, and was finished with a last set of questionnaires (T3) and the last classification of the child’s trauma symptoms according to the DC: 05. The classification of trauma symptoms according to the DC: 05 was in some cases delayed as compared to the completion of the questionnaire, because this could only be done during an appointment between the therapist and family. Also, similar circumstances as mentioned for the EMDR sessions, such as illness of the infant, were sometimes present for these appointments.

### Intervention

EMDR Storytelling (De Roos & Beer, 2017; Lovett, 1999) is already applied in young infants by PH, AD and IS. The goal of this treatment is to help infant process disturbing memories and experiences which presumably underlie the complaints. The underlying idea is that infants can, through various daily triggers, also be reminded of painful experiences. By placing these experiences in a (narrative) context while the infant’s working memory is being taxed by EMDR techniques (e.g., tapping), the memories can be stored more adaptively and less emotionally charged. Stagnated infant development can, in this way, get back on track. The parents can, by writing the story, first reflect on how the child presumably experienced or still experiences the stressful event(s), and then provide the support to the child during the procedure that they were unable to provide at the time of the event. Parents are supported in writing the story in one or two sessions during the baseline phase. The actual EMDR sessions take 45–60 min each. At the start of these sessions, the infants get used to the therapist and the room, and then to the EMDR techniques. The family receives some information about these techniques and that they can be experienced as intrusive, and that the therapist will look for a good way to apply them. The therapist may also use a plush toy or other things the infant may find interesting. When the actual Storytelling starts, the infant is sitting on the lap of one of the parents. After a relaxing moment with the infant, one parent reads the traumatic story to the infant while the therapist provides tapping of the infant’s feet, hands or legs. The tapping, which taxes the working memory of the infant, may be done on places of the body where the traumatic events took place, but only if the parents and the therapist assume and observe that the baby does not experience it as intrusive. Other trauma-related stimuli to activate the trauma memory are also used such as audio-files with sounds of the intensive care, and medical objects, which may be shown during the reading of the story. The reading of the story is alternated with the parents and infant doing something relaxing (i.e., sing a song together). All EMDR Storytelling treatments took place in the period between July and December 2022, and were given by PH and one of her colleagues at OKL. Both were experienced in working with infant and their parents in general and with offering EMDR Storytelling specifically.

### Outcome Measures

#### Treatment Feasibility

To assess feasibility of EMDR Storytelling, we counted the number of participants that completed the full EMDR Storytelling treatment.

#### Treatment Acceptability

To assess acceptability of EMDR Storytelling, parents completed the 8-item shortened version (Nguyen et al., 1983) of The Client Satisfaction Questionnaire (CSQ-8; Attkisson & Zwick, 1982) at follow-up. The items of the CSQ-8 are scored on a scale from 1 to 4, with higher scores indicating a higher level of satisfaction. An example of an item is: “How satisfied are you with the amount of help you have received?” The CSQ-8 has shown to have good psychometric properties (De Brey, 1983; De Wilde & Hendriks, 2005).

#### PTSD Diagnosis Infant

The Dutch translation (Zero to Three, 2019) of the Diagnostic Classification of Psychological and Developmental Disorders in Infancy and Early Childhood (DC: 05, Zero to Three, 2016) was used by the therapist to check two of the inclusion criteria at T0, namely the presence of PTSD in the child, the absence of a disrupted relationship between parents and child and the presence of medical disorder(s) or condition(s), past or present. PTSD can be scored on axis I of the DC: 05, which contains the diagnostic criteria of clinical disorders. Parent-child relationship functioning can be scored on axis II, and ranges from 1 (good to good enough developing parent-child relationship) to 4 (disturbed to dangerous parent-child relationship). Only children who whose relationships with their parents were classified as level 1 or 2 were included in the study. Preferably two weeks after the second EMDR session, and again three weeks later, two meetings between the therapist and the family took place, in which the criteria of PTSD were checked for the second time and third time, respectively (T2 and T3).

#### PTSD Symptoms Infant

The Infant Trauma-related Stress Symptoms Questionnaire (ITSSQ; Holtkamp, 2020) is a checklist developed by and utilized in the context of clinical practice with EMDR Storytelling by *p*H. It contains 27 items and assesses trauma-related symptoms of distress and dysregulation. The items are scored on a Likert-scale from 1 to 3, and recoded on a scale from zero to 2. An example of an item is ‘My baby’s body is tense’. The checklist has been administered at T0, T1, T2, and T3. Because the limited number of participants, interrater reliability was calculated on the basis of all measurement occasions and was shown to be good (Crohnbach’s alpha = .82).

PTSD symptoms of the infant were also measured with personalized items, that were administered daily during the baseline, the intervention and follow-up phase. On the basis of the information from the intake, and hypotheses about what symptoms were related to the traumatic events, and in cooperation with one of the parents, personalized items were constructed to measure the intensity of the existing symptoms throughout the study phases. A 7-point Likert scale was used for all personal items.

### Statistical Analyses

A mean score and standard deviation were calculated of the sum score(s) of the CSQ-8 and ITSSQ. The visual and quantitative analyses of the personalized items that were administered daily were done with the Shiny App for Single-Case Data Analysis (Shiny SCDA V2.8; De et al., 2020). We made graphs depicting the scores of each item during all three phases. We looked at the average mean scores of the baseline, the intervention, and follow-up phase, we compared the average mean scores between the phases, and looked at the slope of the trend lines during the phases. Also, for each personalized item, the ‘nonoverlap of all pairs’ (NAP-) scores were calculated for the combination of all phases: baseline and intervention phase, intervention and follow-up phase, and baseline and follow-up phase. NAP quantifies in how far the scores of one phase match with or differ from the scores of another phase (Parker & Vannest, 2009). Each data point in one phase is being compared to each data point of another phase (the same, higher or lower). A NAP-score is the proportion of non-overlapping data points between the phases. The NAP-score is between the zero and 1, and a score >.5 suggests an effect in the expected direction, which, in our study was a decrease in symptoms between baseline phase and intervention phase, and between baseline phase and follow-up phase.

## Results

### Feasibility and Acceptability

A description of the (medical) background of the infants and of the responses of the infants to the treatment is given in the Supplementary materials. All families finished the EMDR treatment, and all completed the Client Satisfaction Questionnaire at T2. The average mean score of the CSQ-8, which was scored on 4-point Likert scale, was 3.7 (sd = .3). All parents were either satisfied or very satisfied with the intervention. Other results are displayed in Table 1.

**Table 1.** Evaluation of EMDR Storytelling Using the 8-Item Client Satisfaction Questionnaire

Question	Response options			
	Bad	Reasonable	Good	Excellent
What do you think of the quality of EMDR storytelling	–	–	1 (16.7%)	5 (83.3%)
	Certainly not	Hardly	Generally yes	Yes, definitely
Did you receive the kind of help you were hoping for?	–	–	2 (33.3%)	4 (66.7%)
Would you recommend EMDR storytelling to friends?	–	–	–	6 (100%)
	None of my wishes are fulfilled	Only some of them are fulfilled	Most of them are fulfilled	All my wishes are fulfilled
In how far did EMDR storytelling fulfill your wishes?	–	1 (16.7%)	2 (33.3%)	3 (50.0%)
	Very dissatisfied	Dissatisfied	Satisfied	Very satisfied
How satisfied are you about the quantity of help you received?	–	–	–	6 (100%)
How satisfied are you in general with EMDR storytelling	–	–	2 (33.3%)	4 (66.7%)
	It made it worse	Hardly	It did help	Very much
In how far did EMDR storytelling help with your infant’s symptoms?	–	1 (16.6%)	2 (33.3%)	3 (50.0%)
	Certainly not	I don’t think so	I do think so	Yes, for sure
Would you do EMDR storytelling again, if needed?	–	–	1 (16.7%)	5 (83.3%)

### Infant Trauma-related Stress Symptoms and PTSD Diagnosis

**Figure 1.**
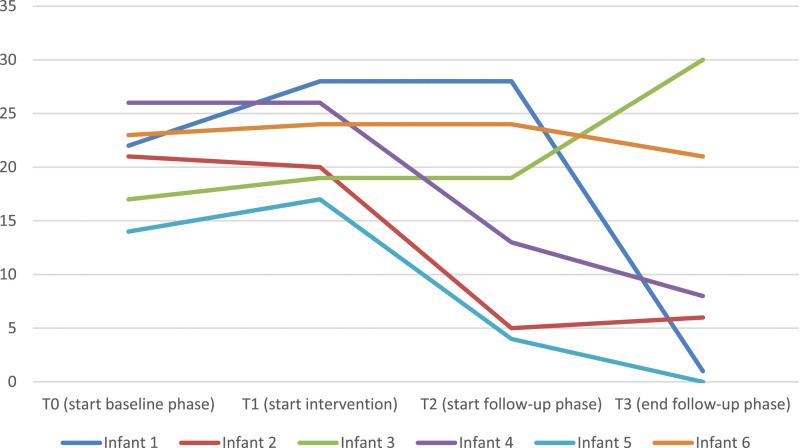
The ITSSQ Scores of Each Infant at T0, T1, T2 and T3. For the Two Missing Items of Infant 1 and 3 at T2, the Scores Were Imputed Using the Last Observation Carried Forward

The sum scores on the ITSSQ of each infant at T0, T1, T2 and T3 are displayed in Table 2 and in Figure 1. For the Figure, missing scores were imputed with the last observation carried forward method. Visual analysis of the figure shows that four children show a clear decrease in symptoms at T3, while one child shows a clear increase and for one child there is no increase and no decrease in symptoms. The presence of a PTSD diagnosis at T0, T2 and T3 are also displayed in Table 2. At T0, all six infants were diagnosed with PTSD. At post-test, only two children of the six children (33.3%) did not have PTSD anymore, according to the DC: 05. At follow-up, in five of the six (83.3%) children, the DC: 05 was used again to classify the infants’ symptoms. All five children were no longer diagnosed with PTSD. The outcomes per child are displayed in Table 2.

**Table 2.** Infant Trauma Symptoms Scores on T0, T1, T2, and T3, and Reliable Change Index (RCI) of Change at T1, T2, and T3 as Compared to T0

	ITSSQ sumscore				PTSD diagnosis		
Infant	T0	T1	T2	T3	T0	T2	T3
1	22	28	–	1	PTSD	PTSD	No PTSD
2	21	20	5	6	PTSD	No PTSD	No PTSD
3	17	19	–	30	PTSD	PTSD	No PTSD
4	26	26	13	8	PTSD	PTSD	No PTSD
5	14	17	4	0	PTSD	No PTSD	No PTSD
6	23	24	24	21	PTSD	PTSD	–

*Note.* T0 Baseline, T1 Pretest, T2 Posttest, T3 Follow-up. The intervention is given between T1 and T2.

### Visual Analysis of Personalized Trauma Symptoms

Daily measurements of personalized trauma symptoms were available for infant 1, 2, 4, 5 and 6. The items of infant 1 concerned crying, overstretching, looking fearful and physical restlessness. The items of infant 2 concerned crying, waking up during the night, overstretching, and clinging. The items of infant 4 concerned resistance of diaper changes, sleep resistance, crying inconsolably and waking up during the night. The items of infant 5 concerned the need to be held, waking up during the night, the frequency of the demand to be fed, and crying. The items of infant 6 concerned waking up during the night, fear of noises, restlessness, and sleep resistance. The data of the daily measurements of infant 3 was not suitable for analysis due to the low number of data points. When formulating the personalized items of infant 3, the mother said that her infant mainly showed symptoms of fear when confronted with medical personnel. This, however, did not happen often enough during the study period.

**Figure 2.**
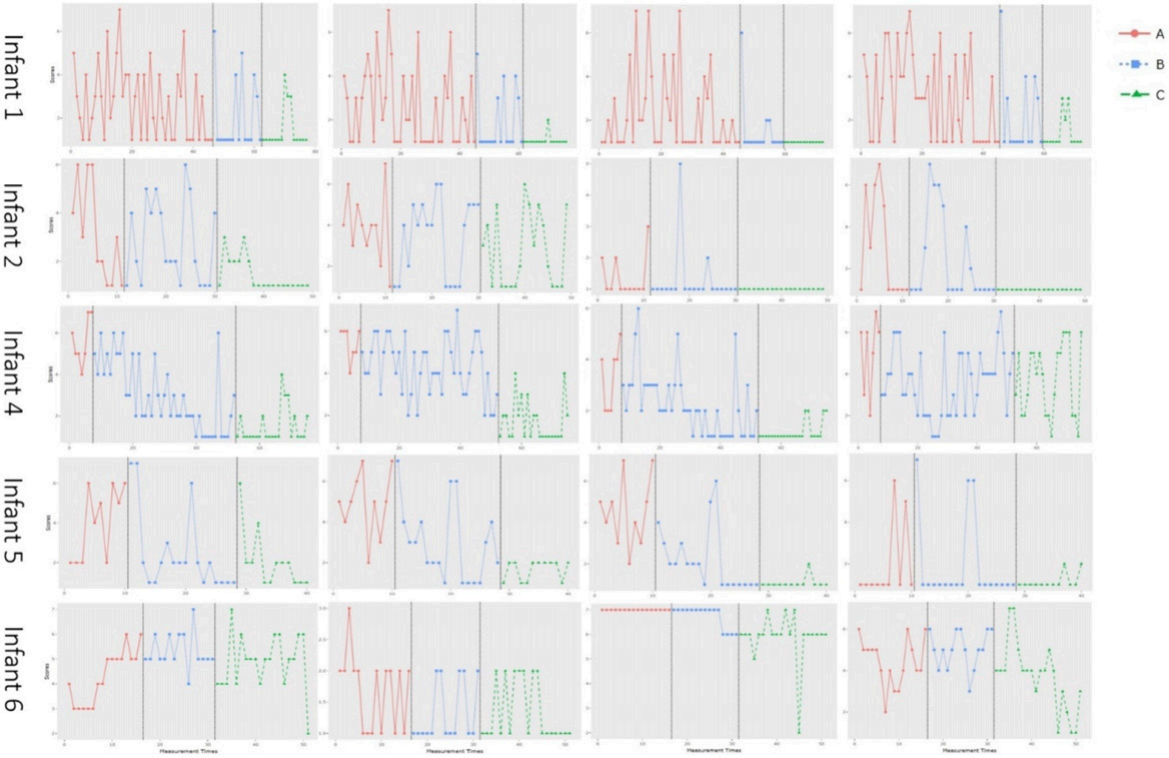
The Graphs of the Scores of the Daily Items that the Parents Completed on Personalized Trauma Symptoms of Their Infant During the Baseline Phase (A), the Intervention Phase (B) and the Follow-Up Phase (C)

Figure 2 depicts the graphs of the scores of the daily items that the parents completed on personalized trauma symptoms of their infant. Mean scores of the personalized items for all infants and each measurement phase, and differences in mean scores between the phases are shown in Table 3. For most items (all items of infant 1, 2, 5 and item 1, 2 and 3 of infant 4), there was a decrease in mean scores from the baseline phase to the intervention phase, and a further decrease in mean scores in the follow-up phase. There were a few exceptions. For item 4 of infant 4, there was a decrease of 2.0 between the baseline phase and the intervention phase, but an increase of 0.1 between the intervention phase and the follow-up phase. For infant 6, the direction of the differences was inconsistent. Looking at the differences between the baseline phase and the follow-up phase, there was an increase in two of the items, and a decrease in two of the items.

**Table 3.** Mean Scores of the Personalized Items for all Children and Each Measurement Phase, and Differences in Mean Scores Between the Phases

Infant		Item 1			Item 2			Item 3			Item 4		
	Phase	Mean	Dif. w/B	Dif. w/C	Mean	Dif. w/B	Dif. w/C	Mean	Dif. w/B	Dif. w/C	Mean	Dif. w/B	Dif. w/C
1	A	2.7	0.6	1.2	2.6	0.7	1.5	2.7	1.3	1.7	2.7	0.6	1.3
	B	2.1	–	.06	1.9	–	0.8	1.4	–	0.4	2.1	–	0.7
	C	1.5	–	–	1.1	–	–	1.0	–	–	1.4	–	–
2	A	3.2	0.4	1.8	3.9	0.5	1.2	1.4	0.1	0.4	3.2	0.8	2.2
	B	2.8	–	1.4	3.4	–	0.7	1.3	–	0.3	2.4	–	1.4
	C	1.4	–	–	2.7	–	–	1.0	–	–	1.0	–	–
4	A	5.6	2.6	4.1	5.4	1.1	3.7	5.6	3.4	4.4	5.6	2.0	1.9
	B	3.0	–	1.5	4.3	–	2.6	2.2	–	1.0	3.6	–	−0.1
	C	1.5	–	–	1.7	–	–	1.2	–	–	3.7	–	–
5	A	4.0	1.6	1.9	4.9	2.0	3.2	4.0	1.8	2.9	4.0	2.1	2.8
	B	2.4	–	0.3	2.9	–	1.2	2.2	–	1.1	1.9	–	0.7
	C	2.1	–	–	1.7	–	–	1.1	–	–	1.2	–	–
6	A	4.3	−1.0	−0.6	1.7	0.4	0.3	4.3	−2.4	−1.6	4.3	−0.7	0.6
	B	5.3	–	0.4	1.3	–	−0.1	6.7	–	0.8	5.0	–	1.3
	C	4.9	–	–	1.4	–	–	5.9	–	–	3.7	–	–

*Note.* Dif. w/ means Difference with. A Baseline phase, B Intervention phase, C Follow-up phase. Positive difference scores indicate a decrease over time, while negative difference scores indicate an increase over time.

Trend lines of the items of all children can be found in the Supplementary materials. The direction of the slopes of the trend lines are summarized in Table 4. The slope of the trend lines in the intervention phase were as expected negative in 15 of the 20 items (75%), which means that there was a decrease in symptoms during the intervention phase. In 9 out of these 15 items (60%), the slope of the trend line was positive or horizontal in the baseline phase, which means that the negative trend during the intervention phase may be attributed to the intervention. However, in 6 of the 15 items that had negative trend lines during the intervention phase, the trend line was already negative during the baseline phase, which makes it unlikely that the decrease during the intervention phase can be attributed to the training. Regarding the follow-up phase: in three of the 20 items (15%), the symptoms was completely absent in this phase. In eight items (40%), the trend lines were positive during the follow-up phase (increasing symptoms) and in seven items (35%) the trend lines were negative (decreasing symptoms).

**Table 4.** Direction of Slopes in the Trend Lines in the Baseline (A), Intervention (B) and Follow-Up Phase (C) of all Items of all Babies

Infant	Item 1			Item 2			Item 3			Item 4		
	A	B	C	A	B	C	A	B	C	A	B	C
1	−	−	+	−	+	+	−	−	0	−	−	−
2	−	−	−	−	+	+	+	−	0	−	−	0
4	+	−	+	−	−	−	+	−	+	+	+	+
5	+	−	−	+	−	+	+	−	+	+	−	+
6	+	−	+	−	+	−	+/−	−	−	−	+	−

*Note.* + positive trend line, indicating an increase in symptoms during the phase, − negative trend line, indicating an decrease in symptoms during the phase, +/− horizontal trend line, zero symptom absent during the phase.

### Quantitative Analysis of Personalized Trauma Symptoms

For each personalized item, the NAP-scores were calculated for the combination of all phases: baseline and intervention phase, intervention and follow-up phase, and baseline and follow-up phase. NAP-scores are presented in Table 5. All effects except for some of infant 6 are in the expected direction. For infant 1, the effects between the baseline phase and the intervention phase, and between the intervention phase and the follow-up phase were weak. However, the effects between the baseline phase and the follow-up phase were moderate. The same was the case for infant 2, with the exception of the effect of item 1 between the intervention phase and the follow-up phase, which was moderate, and the effect of item 3 between the baseline phase and the intervention phase, which was weak. Infant 4 had moderate effects between the baseline and intervention phase. The effects between the intervention phase and follow-up phase ranged from absent to large. The effects between the baseline phase and follow-up phase were large for three items and moderate for the fourth item. Infant 5 showed moderate effects between the baseline phase and the intervention phase for three items, and for the fourth item, the effect was absent. The effects between the intervention phase and the follow-up phase ranged from absent to moderate. The effects between baseline and follow-up ranged from absent for one item to large for two items. Infant 6 showed mixed results with both negative and positive effects, which were mostly weak to moderate.

**Table 5.** Nonoverlap of all Pairs (NAP-)scores for the Combination of all Phases

Infant	Item 1			Item 2			Item 3			Item 4		
	AB	BC	AC	AB	BC	AC	AB	BC	AC	AB	BC	AC
1	**.63**	**.58**	**.72**	**.62**	**.63**	**.76**	**.64**	**.61**	**.73**	**.64**	**.59**	**.74**
2	**.56**	**.74**	**.77**	**.54**	**.60**	**.67**	**.58**	**.55**	**.64**	**.56**	**.68**	**.73**
4	**.88**	**.79**	**>.99**	**.75**	**.93**	**.99**	**.73**	**.79**	**.96**	**.73**	.48	**.73**
5	**.74**	**.53**	**.81**	**.79**	**.69**	**.97**	**.86**	**.75**	**>.99**	**.51**	**.51**	**.53**
6	.24	**.61**	.35	**.66**	.49	**.65**	**.63**	**.81**	**.93**	.36	**.77**	**.69**

*Note.* A baseline phase, B intervention phase, C follow-up phase.

NAP <.50 effect opposite of the expected direction, NAP >.50 (bold) effect in the expected direction.

.50 < NAP <.65 weak effect, .66 < NAP <.92 moderate effect, .93 < NAP <1.0 strong effect.

## Discussion

The aim of this pilot study was to assess the feasibility, acceptability, and initial indications of effectiveness of a 2-sessions EMDR Storytelling intervention for infants aged zero–24 months. The 6 infants that were included in the current study had a background of admissions in the hospital and medical procedures. Some of the infants were admitted to the NICU right after birth, while others were hospitalized after a few days, weeks or months, and one infant around his first birthday. The conditions the infants suffered from ranged from perinatal asphyxia, infections and inflammations, failure to thrive, chromosomal syndrome, pseudocroup, choking because of vomiting, and heart conditions. Some of the infants needed to be operated, while others underwent medical procedures such as scans and blood tests. All infants whose parents completed the daily measurements had regulation problems related to crying and sleeping. Furthermore, post-traumatic symptoms such as overstretching, clinging, fearful reactions, and physical restlessness were reported.

We found that EMDR Storytelling was indeed feasible (all families completed the intervention) and acceptable (all parents were either satisfied or very satisfied with the intervention). Visual analysis indicated that, at the last measurement occasion, the follow-up measurement, which was done 5 weeks after the last EMDR session, four out of the six infants showed an improvement in parent-rated trauma symptoms, one did not show any change, and one showed a deterioration, and five out of the six infants were no longer classified with a post-traumatic stress disorder. Parents of five of the six infants completed the daily measurements of the items on personalized symptoms. The parents of all children reported a moderate or large effect sized improvement on at least one item between the baseline phase and the follow-up phase, and for four of the 5 children, this was the case for at least three of the four items. However, based on the trend lines of the items at the baseline and intervention phases, only in about half of the items in which improvement was shown, the improvement may be attributed to the intervention. There was no clear consistency in the kind of items that showed improvement during the intervention phase but not during the baseline phase. All in all, four of the six infants included in the current study showed a clear reduction in PTSD symptoms over time. The results concerning the other two infants were contradictory. In these children, other problems than the PTSD-symptoms only seemed to play a role: infant 3 had a syndrome that caused regulation problems and there were unsolved family problems in the family of infant 6. The parents of infant 3 did indicate that they had the impression that EMDR had had a very positive impact on infant 3, especially on the way she was able to deal with medical personal.

Although EMDR Storytelling has been used by infant mental health specialists and EMDR practitioners with children below 2 years old, and beneficial results were observed, there were no studies available on the benefits of this intervention in this specific age group. The current study shows that the intervention seems to be feasible for families with and infant with PTSD symptoms after undergoing medical procedures, even during the first 6 months of their life. All parents were able to write a story together with the therapist, although one mother needed trauma treatment for herself first, before being able to write and read the story to her infant. The EMDR sessions were carried out according to the plan; the reading of the story was alternated with moments of relaxation, and all parents and infants attended both sessions, although some sessions needed to be postponed due to for example illness of the infant. Four of the infants showed an emotional reaction to the story in one or both of the sessions. Two of the infants showed signs of processing their trauma via play right after the sessions, for example by diaper changing all stuffed animals in the room. All in all, EMDR Storytelling seems to be a feasible intervention for infants aged less than 24 months with symptoms of post-traumatic stress after being confronted with different medical problems and undergoing different medical procedures. This is in line with the results of a meta-analysis of drop-out in trauma-focused therapies and non-trauma focused treatments in children and young people, showing similar drop-out rates, which lead to the conclusion that trauma-focused therapies seem to be well tolerated in children and young people (Simmons et al., 2021).

The acceptability of EMDR Storytelling was measured with the CSQ-8. The average means score on the CSQ-8, which is scored on a 1 to 4 Likert scale, was 3.7. Parents of two infants were satisfied (33.3%) and four were very satisfied (66.7%) with the intervention. The quality of EMDR Storytelling was rated as good by the parents of one infant (16.7%), and excellent by the parents of five infants (83.3%), and all parents (100%) would recommend EMDR Storytelling to friends. The parents seemed to experience the sessions intense on the one hand, but as a relief on the other hand. Two of the infants showed an initial worsening of their complaints right after the first session, but these symptoms decreased within one or a few days. In sum, the acceptability was high, as reported by the parents of the infants in the current study.

PTSD-classification and -symptoms of the infants were measured using the DC:03 (therapist reported), and the ITSSQ (reported by the parents). Only the last was measured twice before the actual start of the intervention (baseline and pretest). All in all, looking at the combination of therapist-reported PTSD-classification and parent-reported PTSD-symptoms at follow-up, four of the six infants (66.7%) seemed to have benefited from the EMDR Storytelling, while one infant did not seem to have improved (16.7%), and for one infant, the results contradicted each other (16.7%). There are no studies on EMDR Storytelling in infants or even in toddlers available to compare the results of the current study with. However, a study on EMDR in children aged 4–8 years with PTSD was available. In this study, EMDR (and EMDR Storytelling for the children for whom the traumatic event happened before the age of 4) was effective in reaching diagnostic remission in 85.7% of the children (Olivier, de Roos, & Bexkens, 2021). Possibly, the somewhat larger remission rate in that study may be explained by the higher treatment dose (six sessions). Also, some trauma-symptoms in infants such as crying may be less specific for trauma, and may also be symptomatic of other problems in infants, or even of problematic circumstances (such as family problems, a new illness, etc.), making it more difficult to show clear and longer lasting improvements.

PTSD-symptoms of the infants were also measured with personalized items that were scored daily by parents of five of the six participating infants (four per child, thus 20 items in total). Analysis of these items was done both visually and quantitatively. For the visual analyses, graphs depicting the scores during the baseline, intervention, and follow-up phase were made. We compared the mean scores of each item between the phases. For most items, the differences were largest between the baseline phase and the follow-up phase. This confirms the results of the PTSD-classification and symptoms which also suggested that the time it took for symptoms to decrease lasted beyond the intervention phase. For four of the five infants (infant 1, 2, 4 and 5), there was a decrease in symptoms between baseline and follow-up for all items. For the last infant (infant 6), there was a decrease between baseline and follow-up for two items, but also an increase for two other items. These results also confirmed the results of the PTSD-classification and symptoms, which showed clear improvement in infant 1, 2, 4 and 5), but no improvement in infant 6. The improvements that were shown in the current study are in line with the anecdotal evidence on the effectiveness of EMDR Storytelling in infants (Lovett, 1999; Went, 2014).

We also looked at the slope of the trend lines. We expected a negative trend line (which means a decrease in symptoms) during the intervention phase, which was found in 15 of the 20 items in total. However, only a negative trend during the intervention phase in combination with the absence of a negative trend during the baseline phase suggests that the decrease in symptoms during the intervention phase may be attributed to the intervention. This was the case in 9 out of the 15 items that showed a decrease during the intervention phase. These items concerned overstretching (infant 2), resistance of diaper changes and crying inconsolably (infant 4), the need to be held, waking up during the night, the frequency of the demand to be fed, and crying (infant 5), and waking up during the night and restlessness (infant 6). In infant 1, the trend line during the baseline phase was already negative. The baseline phase of this infant was really long due to circumstances, where the need arose to give the mother EMDR treatment herself before the EMDR Storytelling could be started. Possibly, the treatment of the mother may have had a positive impact on the mother-infant relationship and the infant’s symptoms. In infant 2, three of the four trend lines during the baseline were also already negative. During the baseline phase, parents write the story, which helps them to give words to both their own and their infant’s experience. Possibly, this already influences the parent-infant interaction, and the co-regulation between parents and infant, and may therefore also affect the symptoms of the child. The results concerning the trend lines suggest that we need to be careful in the interpretation of the positive findings regarding the differences in the average mean scores of the personalized items between the phases, and the PTSD-classification and –symptoms at follow-up. Although we did show decreases in symptoms over time, this may not be attributed to EMDR Storytelling for all items and for all infants.

We quantitatively analyzed the personalized items by calculating NAP-scores between the baseline and intervention phase, between the intervention phase and the follow-up phase, and between the baseline phase and the follow-up phase. These scores show the direction and the magnitude of an effect. The direction of the effects was as expected for all items of all infants, except for infant 6, whose results were mixed. The only other study evaluating EMDR Storytelling in children that also calculated NAP-scores was a study in children aged 4–12 years who endured child abuse and neglect (VanTieghem et al., 2017 2012). In this study, only 2 out of 8 children showed improvement in their trauma symptoms. Possibly, the nature of the traumatic events made treatment of trauma symptoms more difficult in this group of children.

The current study was the first empirical study evaluating EMDR Storytelling in infants aged zero–24 months. The youngest infant that was admitted to the study was only 4 months at the time of inclusion. Even this very young infant showed a clear reduction in symptoms after the treatment. Earlier empirical studies on the effectiveness of trauma-focused treatment in young children included children aged 2 or 3 and older (Rachamim et al., 2015, 2021; Scheeringa et al., 2011). A strength of the present study was that a combination of measures was used, namely an acceptability questionnaire, therapist-reported classification of PTSD-symptoms, parent-reported PTSD-symptoms, and personalized items that were completed daily by the parents.

The current study also had limitations. The first was the that the length of the baseline phase, intervention phase and follow-up phase was not randomized. Instead, the intention was to standardize the length of all phases, but in practice, the length was often determined by circumstances such as illness of the infant or therapist, or the need to give the parents EMDR treatment first, before being able to start the EMDR treatment of the child. These circumstances may have influenced not only the length of the phases, but also the treatment trajectories and their outcomes. Also, the questionnaires of T2 and T3 were completed later than planned for two children. This was a methodological limitation of the study. We administered questionnaires on sensory problems and parental responsiveness/parent-infant co-regulation, but due to a very large percentage of missing questionnaires, we were not able to include the data in the current article. In future studies, these concepts could be included. Because the DC: 0-5 classification of PTSD was only administered at T0 (start of the baseline period), T2 (end of the intervention period), and T3 (end of the follow-up period), but not at T1 (end of the baseline period), we do not know whether there were any infants who would have no longer been classified with PTSD at the end of the baseline period. This question is relevant for two of the children, who changed from ‘PTSD’ to ‘no PTSD’ between T0 and T2.

Another important limitation was that neither the therapist nor the parents were blind to the treatment status, and both were engaged with, and invested a lot in the treatment process. As the parents and therapists were also the only informants in the current study, this may have biased the results. Hope for improvement and cognitive dissonance may for example have played a role in possible bias. The involvement of the parents in the writing and reading of the story may have had another unintended consequence. We cannot exclude the possibility that this may have been a form of exposure therapy for the parents and that the improvement in possible trauma symptoms of the parent may have been one of the working mechanisms for improvement of the trauma symptoms in the infants. We did not measure PTSD-symptoms in parents, and were therefore not able to study this factor as a potential working mechanism. The question is, however, whether this possible working mechanisms would have a large enough effect in reducing PTSD-symptoms in infants. If the writing of the story would have been beneficial for the children, the positive effects would be visible in the baseline period. Only in about half of the personalized items, there was a declining slope during the baseline phase, while in about the other half, there was an increase in symptoms during the baseline phase. During the intervention phase, in three quarters of the items, we found a declining slope line. Also, the NAP-scores also showed that in 18 of the 20 items, the symptoms were lower in the intervention phase than in the baseline phase. This, together with the illustrations that were given in the case and therapy descriptions, makes us question whether an intervention with the parents only would be effective for most infants.

Furthermore, the design of the current study does not allow us to draw any conclusion on the efficacy of the taxing of the working memory of the infant, as we did not include a phase in which the infant was told the story on the traumatic events, but without the tapping of the infant’s feet, arms or legs. It may be that just telling the story to the infant while the infant is sitting on the parent’s lap may give similar results. Future studies could make use of improved methodological approaches by using randomization of phases, or even by choosing a randomized controlled trial as a research design. Future studies could also use experimental designs with microtrials, that are aimed at determining which element or combination of elements of EMDR Storytelling are effective (Leijten et al., 2015). Examples of elements that could be studied in such a study are for example (1) parental story writing only, (2) reading the story to the parents and not the infant, (3) reading the story while the infant is not being held by the parents, (4) reading the story to the infant without the taxing of the working memory, and (5) reading a neutral story to the infant while taxing the working memory of the infant. Future studies could also include measurements of PTSD-symptoms in parents, so that a decrease in parental PTSD-symptoms can be studied as a potential working mechanism.

## Conclusion

The current study was the first empirical study evaluating EMDR Storytelling in infants who showed symptoms of post-traumatic stress after undergoing medical procedures. The study shows that EMDR Storytelling is feasible and acceptable for infants aged as young as 4 months and their parents. Four of the six infants included in the current study showed a clear reduction in PTSD symptoms over time. The results concerning the other two infants were contradictory. The design of the current study, and the fact that some infants already showed some improvement on the daily personalized items during the baseline phase, limit definite conclusions about the beneficial effects of EMDR Storytelling. Nevertheless, the preliminary results are encouraging. The current study provides initial evidence for the effectiveness of the EMDR Storytelling for infants suffering from PTSD symptoms.

## Supplemental Material

Supplemental Material - Improving Infant Mental Health: A Pilot Study on the Effectiveness, Acceptability and Feasibility of EMDR Storytelling in Infants With Post-traumatic Distress After Medical Procedures Supplemental Material for Improving Infant Mental Health: A Pilot Study on the Effectiveness, Acceptability and Feasibility of EMDR Storytelling in Infants With Post-traumatic Distress After Medical Procedures by Eva Potharst, Petra Holtkamp, Lily Walliser, Agnes H. Dommerholt, Maartje E. N. van den Heuvel, Indra Spierts, and Marija Maric in Evaluation & the Health Professions
