# Supplementary material for: Improving Infant Mental Health: A Pilot Study on the Effectiveness, Acceptability and Feasibility of Eye Movement Desensitization and Reprocessing (EMDR) Storytelling in Infants With Post-traumatic Distress After Medical Procedures
Source: Eval Health Prof. 2024 Oct 23;48(1):55–70. doi: 10.1177/01632787241268176 (PMC11909792; doi:10.1177/01632787241268176)
Supplement: Supplemental Material - Improving Infant Mental Health: A Pilot Study on the Effectiveness, Acceptability and Feasibility of EMDR Storytelling in Infants With Post-traumatic Distress After Medical Procedures [file sj-pdf-1-ehp-10.1177_01632787241268176.pdf]

Supplementary materials

Descriptive responses of children during the treatment

Infant 1 is a 4 months old girl at the time of admission for the EMDR treatment. The birth of this girl and her twin sister at 36 weeks was complicated and her APGAR scores were 1, 3 and 6. She was brought to an academic hospital, where she was treated with therapeutic hypothermia because of perinatal asphyxia. After she was discharged from the neonatal intensive care unit, and was hospitalized in the high care unit, she had an umbilical cord infection. All this time, she was separated from her twin sister, who stayed in the regional hospital after birth. A week after the release from the hospital, she nearly suffocated after vomiting. After recovering, she still seemed physically uncomfortable and she was restless, seemed fearful, overstretched, had sleeping problems and cried a lot.

Although the parents did not seem to have PTSD themselves when this was checked prior to the study inclusion, it became clear that the mother had too many PTSD symptoms to be able to read the EMDR Storytelling story to her infant. She was offered EMDR for herself, which took place in the same treatment center. She also suffered from depressive complaints, for which an adjustment in pharmacological treatment was made. In the same period, the father suffered from a hernia. Because of these reasons the EMDR sessions with the infant were postponed, and the baseline period was much longer than planned. At the first EMDR session with the infant, she was sitting with the mother, while the father read the story, and the therapist tapped her legs. She was screaming and crying during the reading of the story. After the first session, parents reported that she was calmer at home. The second time, she did not cry any more, but was still physically restless. Parents reported that the trauma complaints diminished after the second session.

Infant 2 is a 22 months old boy when admitted for EMDR treatment. The birth of this infant was uncomplicated at 38 weeks, but soon after his first birthday, he had a bacterial infection in his spinal cord, for which he underwent many painful and fear-inducing medical procedures, such as an insertion of a

brachial artery catheter, during which he woke up from sedation, a nuclear bone scan, and placement of a subclavian catheter. It took long before it became clear what caused the pain and what treatment he needed. His coping was to never resist any procedure, but instead be very friendly to the medical staff. The infection may have caused damage to some of the dorsal vertebrae. Since the infection, the infant developed sleeping problems. He woke up screaming several times a night. Furthermore, he cried long and inconsolably, he overstretched regularly, and clinged to his mother.

During the first session of EMDR, he silently listened to the story with wide open eyes and did not resist, which was similar to how he reacted to medical procedures. The second time he did show a lot of resistance, he screamed, and did not want to stay on his father's or mother's lap. The therapist let him leave the room twice during the session, but he did return to the room, and the story was finished, while he was still screaming. The parents reported that the crying and screaming at home diminished and he fell asleep much easier after the treatment.

Infant 3 is a 14 months old girl at time of admission, who was born very small for her gestational age with 36 weeks. She was diagnosed with failure to thrive, and was assessed medically to find the cause of the failure to thrive. Also, since her birth, she was ill oftentimes. Medical procedures included scans, ultrasounds, x-ray imaging, and blood tests. A specific chromosomal syndrome was found. The main symptom of this infant was a very fearful reaction to people she did not know, and especially to medical staff, and to medical procedures such as measuring of the head growth, which was needed often because of her syndrome, and the use of medical skin stickers. Oftentimes she was restrained by medical staff during these procedures.

The first session, much attention was given to the measuring of the head growth, both by the story that was told, and by showing her a measuring tape. She reacted with much fear, but at the end of the session, she started playing with the measure, and measuring the head of the people in the room. She also played with other objects from the toy doctor's case. The second session, much attention was paid to the drawing of blood and the use of medical skin stickers. The therapist made use of the EMDR buzzers, which the infant seemed to like a lot. After the session, she was given the stickers to put on others and herself.

She played a lot with the stickers and a doctor's case at home after the sessions. When it was needed again to measure her head growth and draw blood, it became clear that she was no longer afraid of these procedures, and it was not needed to restrain her during these procedures. However, her regulation problems did become worse. After the follow-up measurement of the study, it became clear that she had suffered from a combination of sensory issues and an intolerance for dairy products. After the parents made adjustments, for example in her diet, her behaviour improved again.

Infant 4 is a 23 months old girl, who was born with 36 weeks. In the first week after her birth she was hospitalized because of an infection, and received intravenous antibiotics. At six months, she was hospitalized again because of pseudocroup, and at 17 months because of a renal pelvic inflammation. She needed to have her blood drawn several times, which was very painful for her because it was difficult finding a vein. This hospitalisation happened during the COVID-19 pandemic, and therefore, only the mother stayed with her in the hospital, because a second parent was not allowed. In between these hospitalizations, she had bladder infections and renal pelvic inflammations several times, for which she was treated with antibiotics. Because of these inflammations, urine samples needed to be taken regularly. She then seemed to not want to urinate anymore, which worsened the physical problem. She started to show sleeping problems, reacted with anxiety to diaper changes both at home and at the day care centre, was fearful when she saw medical staff, was anxious and withdrawn at the day-care centre, and showed a strong preference for her mother over her father.

During the first session, most attention was given to the inflammations, the medical procedures around the inflammations, and the difficulty and fear she experienced when changing her diaper. She listened to the Storytelling story attentively, and when the EMDR was finished, she started to diaper change all the stuffed animals in the room, and later at home, she changed diapers of all her dolls. After this session, her parents and the carers of the day care started to notice that changing her diaper got easier. The second session was postponed because of another infection that the infant caught, then the family's vacation, and then a COVID-19 infection of the therapist. Therefore, the intervention phase lasted longer than planned. At the hospitalization after the first EMDR, the

parents noticed that she was more willing to let a doctor look at her. After this hospitalization, she received long-term antibiotics to prevent more inflammations. At the second session, extra attention was paid to the separation from her father, and to the explanation for this separation. Also attention was being paid to the pain and fear that she had experienced during nights that she was ill, and to the fact that she did not want to be separated from her mother now. Lastly, attention was being paid to specific words that she reacted to with anxiety (such as ‘ill’ and ‘paracetamol’), as well as medical objects that she reacted to with anxiety, such as thermometers and medical stickers, with which she was allowed to play with at the end of and after this second session. Parents noticed that after this session, she was no longer afraid to receive a medical sticker when she had hurt herself. Also after this second session, the father received advice on how to repair the contact with her, and the contact between them slowly started to improve.

Infant 5 is a 13 months old boy when admitted for EMDR, who was born with 40 weeks. When he was 10 days old, he had an apnea after choking because of frequent vomiting that he suffered from as a young baby. He was brought to the hospital by an ambulance. After this incident, he had a viral respiratory infection and was short of breath, and he panicked when that happened. He developed severe sleeping problems. He woke up at night up to ten times, wanting to drink a lot of water each time. His parents and caretakers of the day-care centre observed much tension, and he showed signs of separation anxiety. He was whining a lot.

The parents and the therapist wrote a story on the incident, but also on having a shortness of breath when he had a viral respiratory infection, and how this reminded him of the incident. The first EMDR session, the infant started out sitting at his mother’s lap while the father read the story. He listened but did not react. Then the parents switched roles, and the mother was emotional while reading the story. The infant became uneasy and wanted to sit with his mother, and he cried a little on her lap. During the second session, he did not react to the story. However, he did start to sleep much better, and when he started to sleep

through the night, he also started to take developmental steps. He was much happier, also at the day care centre. He did no longer react with fear to having a cold.

Infant 6 is a 9 months old boy who was born with 38 weeks. At the 20-week ultrasound, a heart disease was diagnosed, namely a transposition of the great arteries. He had a difficult start after birth, and had many medical complications after the heart operation. During the operation, another heart condition was found, namely major aortopulmonary collateral artery. He received percutaneous coronary intervention twice. His parents were not allowed to hold him during this first period of his life. When the infant was released from the hospital, he had sleeping problems, he cried often, and was sensitive to sounds. He was breastfed, and woke up several times a night to be fed. He was somewhat avoidant in contact with the parents.

The parents and the therapist wrote a story about the operation and medical procedures. The story was read during two EMDR sessions with both parents. The infant reacted with resistance. When the story was read, the infant calmed down quickly, and started exploring the room. He tended to avoid the father less after the second session. After the EMDR sessions, the mother said that she saw no improvement in the sleeping problems, and that he should receive sleep training, but that neither of the parents had the energy or possibility to do this with him.

Plots with trend lines of the personalized items of baby 1 (on top item 1 and 2, and below item 3 and 4):

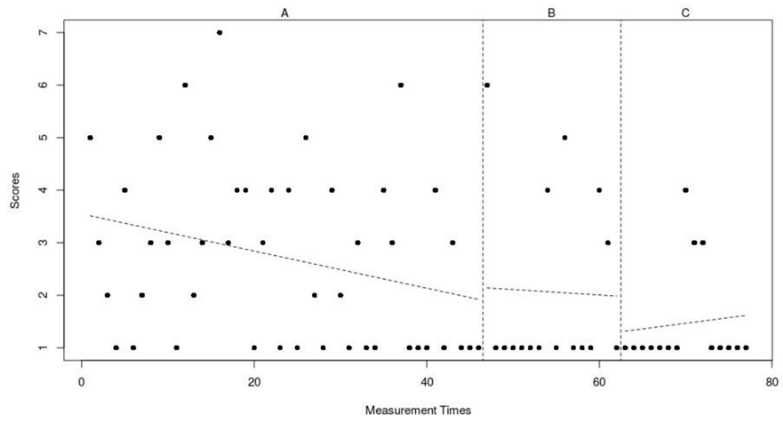

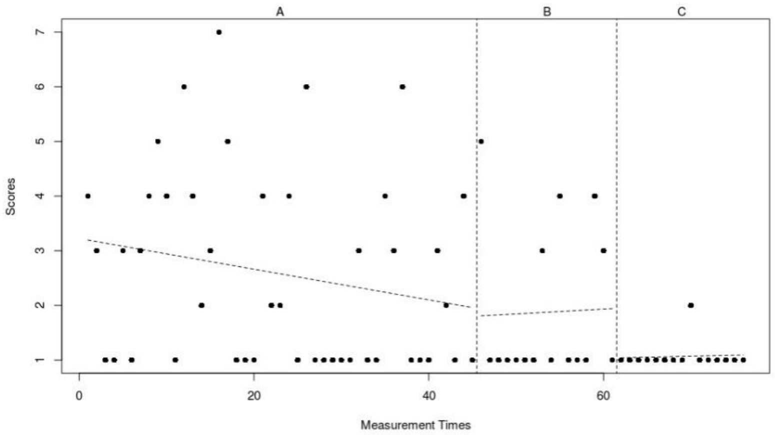

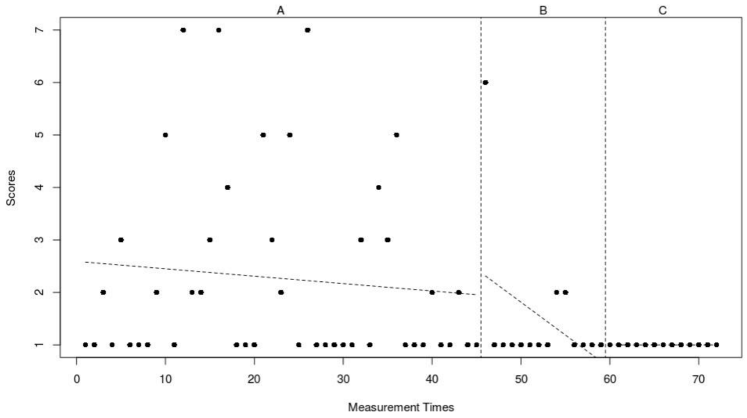

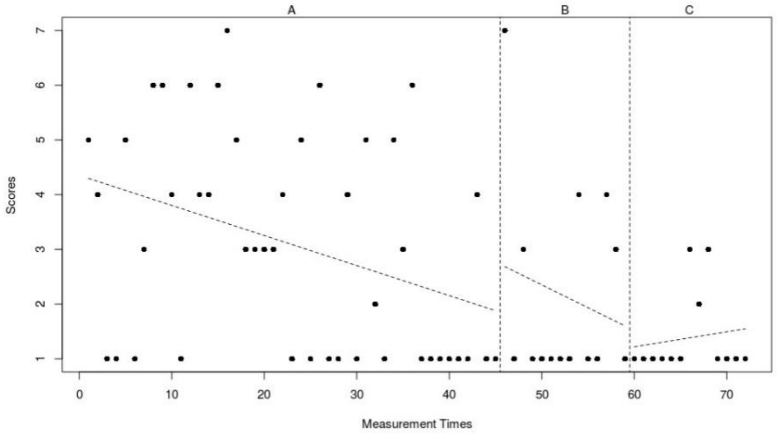

Plots with trend lines of the personalized items of baby 2 (on top item 1 and 2, and below item 3 and 4):

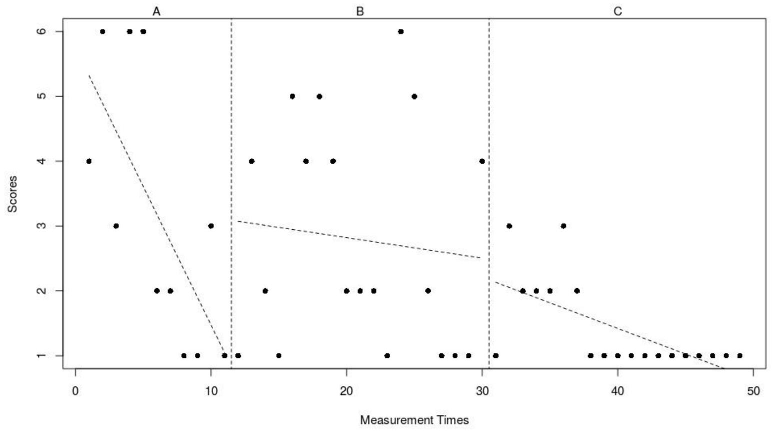

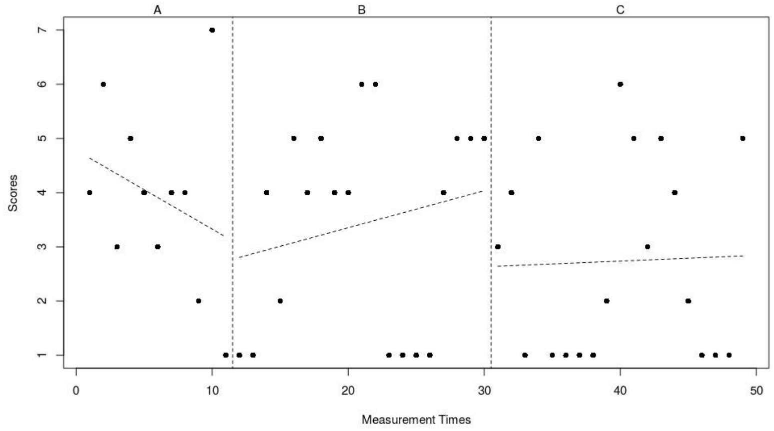

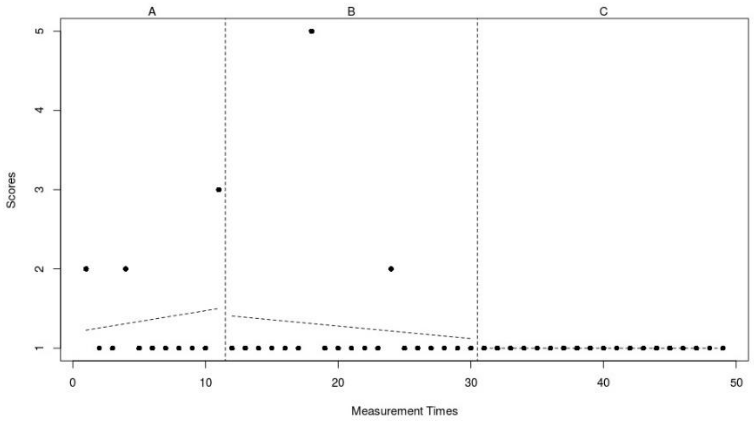

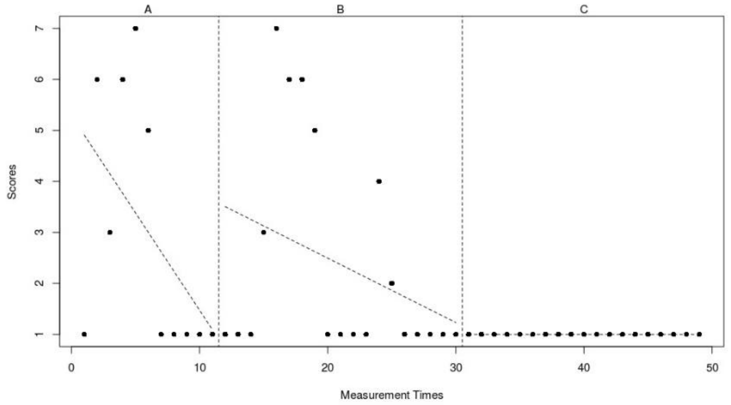

Plots with trend lines of the personalized items of baby 4 (on top item 1 and 2, and below item 3 and 4):

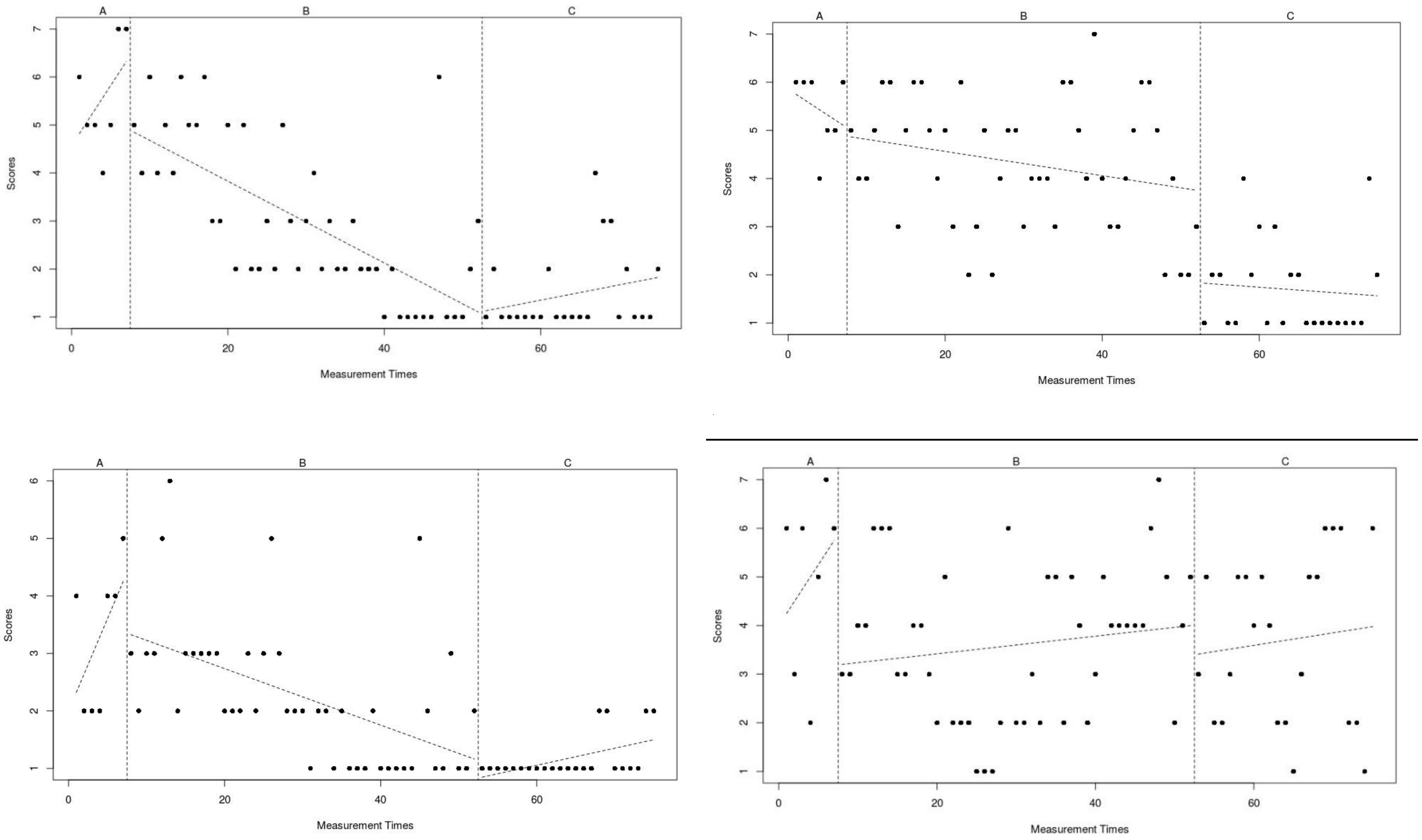

Plots with trend lines of the personalized items of baby 5 (on top item 1 and 2, and below item 3 and 4):

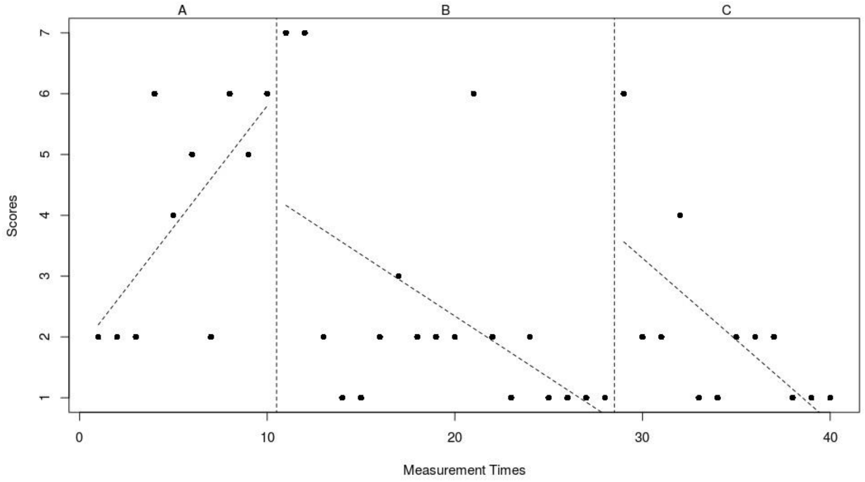

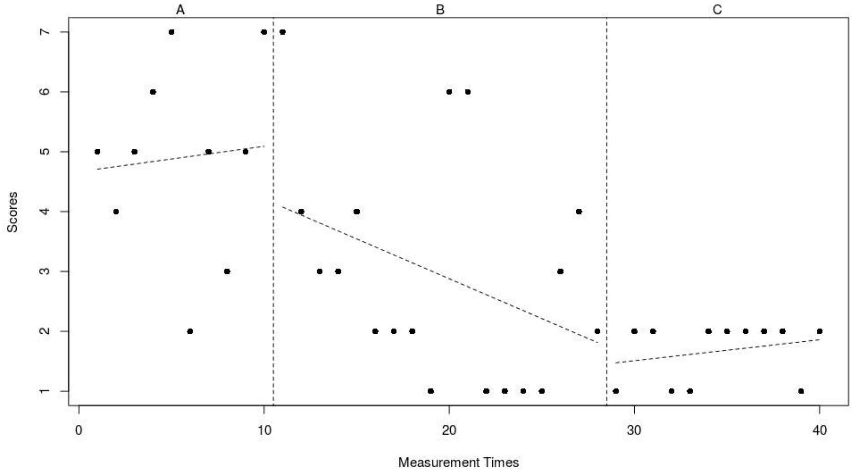

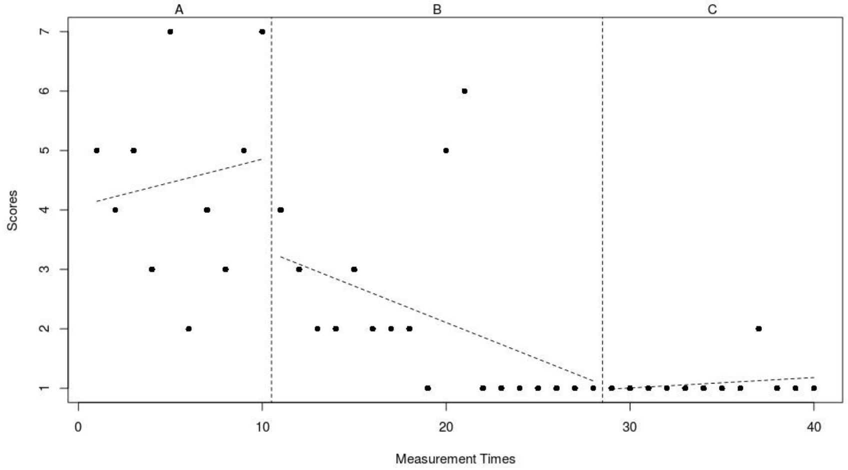

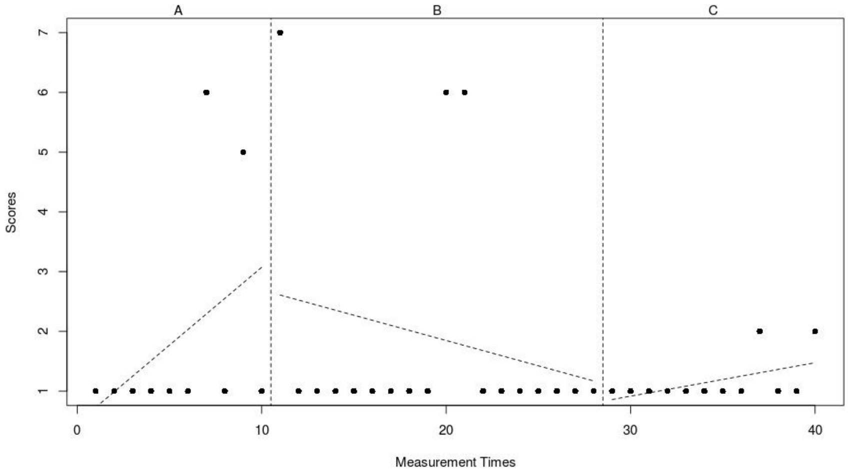

Plots with trend lines of the personalized items of baby 6 (on top item 1 and 2, and below item 3 and 4):

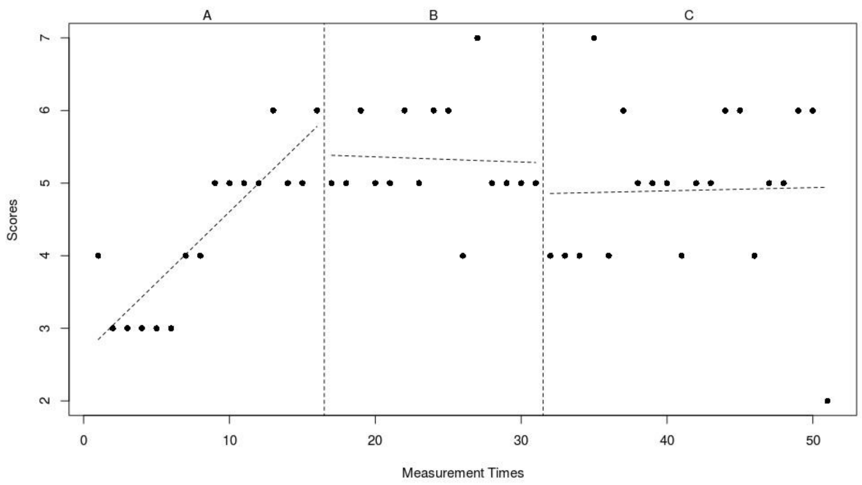
A
B
C
Scores
2
3
4
5
6
7
0
10
20
30
40
50
Measurement Times

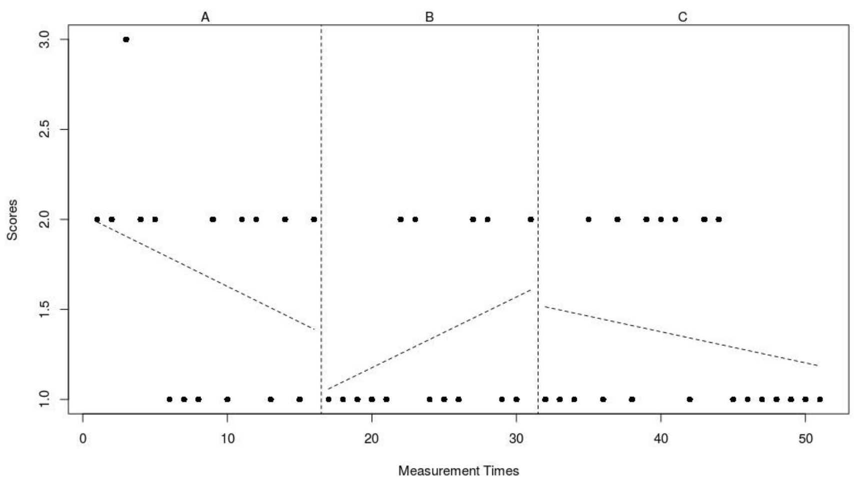
A
B
C
Scores
1.0
1.5
2.0
2.5
3.0
0
10
20
30
40
50
Measurement Times

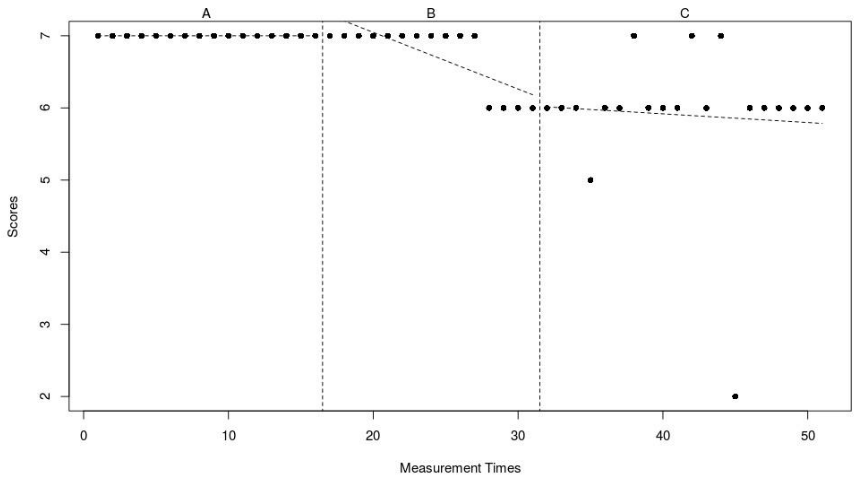
A
B
C
Scores
2
3
4
5
6
7
0
10
20
30
40
50
Measurement Times

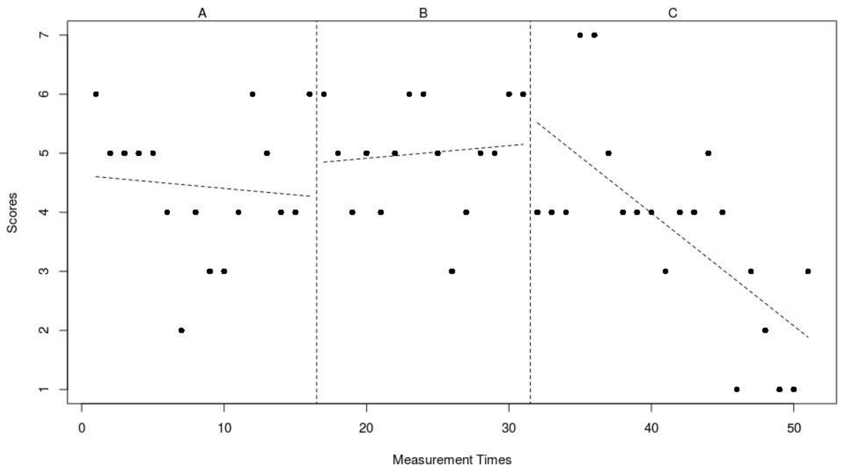
A
B
C
Scores
1
2
3
4
5
6
7
0
10
20
30
40
50
Measurement Times
